# Efficient Preparation of Bafilomycin A1 from Marine *Streptomyces lohii* Fermentation Using Three-Phase Extraction and High-Speed Counter-Current Chromatography

**DOI:** 10.3390/md18060332

**Published:** 2020-06-25

**Authors:** Ye Yuan, Xiaoping He, Tingting Wang, Xingwang Zhang, Zhong Li, Xiaoqing Xu, Weiyan Zhang, Xiaojun Yan, Shengying Li, Shan He

**Affiliations:** 1 Li Dak Sum Yip Yio Chin Kenneth Li Marine Biopharmaceutical Research Center, College of Food and Pharmaceutical Sciences, Ningbo University, Ningbo 315800, China; 23yuanye@163.com (Y.Y.); 18892628896@163.com (X.H.); 18251965896@163.com (T.W.); zhangweiyan13@126.com (W.Z.); 2 School of Marine Science, Ningbo University, Ningbo 315211, China; 3 State Key Laboratory of Microbial Technology, Shandong University, Qingdao 266237, China; zhangxingwang@sdu.edu.cn; 4 Shandong Provincial Key Laboratory of Synthetic Biology, and CAS Key Laboratory of Biofuels at Qingdao Institute of Bioenergy and Bioprocess Technology, Chinese Academy of Sciences, Qingdao 266101, China; lizhong@qibebt.ac.cn; 5 University of Chinese Academy of Sciences, Beijing 100049, China; 6 Rushan Hanwei Biological Science and Technology Co., Ltd., Rushan 264502, China; xiaoqingxu@163.com

**Keywords:** three-phase solvent system, oil removal, liquid–liquid extraction, bafilomycin, HSCCC

## Abstract

An efficient strategy was developed for the rapid separation and enrichment of bafilomycin A1 (baf A1) from a crude extract of the marine microorganism *Streptomyces lohii* fermentation. This strategy comprises liquid−liquid extraction (LLE) with a three-phase solvent system (*n*-hexane–ethyl acetate–acetonitrile–water = 7:3:5:5, *v*/*v*/*v*/*v*) followed by separation using high-speed counter-current chromatography (HSCCC). The results showed that a 480.2-mg fraction of baf A1-enriched extract in the middle phase of the three-phase solvent system was prepared from 4.9 g of crude extract after two consecutive one-step operations. Over 99% of soybean oil, the main hydrophobic waste in the crude extract, and the majority of hydrophilic impurities were distributed in the upper and lower phase, respectively. HSCCC was used with a two-phase solvent system composed of *n*-hexane–acetonitrile–water (15:8:12, *v*/*v*/*v*) to isolate and purify baf A1 from the middle phase fraction, which yielded 77.4 mg of baf A1 with > 95% purity within 90 min. The overall recovery of baf A1 in the process was determined to be 95.7%. The use of a three-phase solvent system represents a novel strategy for the simultaneous removal of hydrophobic oil and hydrophilic impurities from a microbial fermentation extract.

## 1. Introduction

Bafilomycin A1 (baf A1) is a 16-membered ring macrolide antibiotic that was first isolated from *Streptomyces griseus* in 1983 [1,2]. Baf A1 was previously reported to show antimicrobial, antiosteoporosis, and anticancer activities [3,4,5]. In particular, baf A1 is potential agent for the treatment of osteoporosis and tumors, because it is an effective inhibitor of both vacuolar H^+^-ATPase (V-ATPase) [3] and sarco/endoplasmic reticulum Ca^2+^-ATPase (SERCA) pumps [6]. However, baf A1 was restricted from becoming a clinical drug due to its marked toxicity to mammalian cells [4]. To lower the toxicity and optimize the pharmacological properties through medicinal chemistry approaches for further drug development, the cost-effective generation of the structurally complex baf A1 supply is important, even though continued efforts on the total synthesis and semi-synthesis of bafilomycin derivatives were made [7,8,9].

In our previous study, a mutant strain marine *Streptomyces lohii* orf2&3 was shown to overproduce baf A1 under optimized culture conditions. During cultivation, this mutant strain required an abundant a feedstock of soybean oil (60 g/L) [10] to be used as a long-term carbon source and a fast polyketide precursor. However, the presence of excess soybean oil complicated the following purification process after it was extracted with organic solvents alongside baf A1 and some other produced natural products from the culture.

As a conventional isolation method, liquid–liquid extraction (LLE) is widely used in crude sample pretreatment due to its low cost, high efficiency, and simple procedure [11,12]. However, the low precision of this method often limits its utility in natural product isolation. In recent years, three-phase solvent systems were successfully applied to the separation of multiple component mixtures with a wide range of hydrophobicity [13,14,15,16,17,18,19]. In principle, a three-phase solvent system is able to form three immiscible phases after settling, consisting of a hydrophobic upper phase (UP), a moderately polar middle phase (MP), and a hydrophilic lower phase (LP) [13]. During extraction, hydrophobic components (such as fatty acids) and hydrophilic ones (such as saccharide, protein) can be well distributed in the UP and LP, respectively [14,15], while intermediate-polarity and drug-like natural products would be enriched in the MP. This method accordingly holds great potential for isolating target molecules from a complex mixture in an efficient manner. To date, several three-phase solvent systems were reported for complex sample pretreatment and further chromatographic isolation, including *n*-hexane–methyl acetate–acetonitrile–water [13,16], *n*-hexane–*tert*-butyl methyl ether–acetonitrile–water [13,17], *n*-heptane–methyl *tert*-butyl ether–acetonitrile–water [18], and *n*-hexane–acetonitrile–dichloromethane–water–ethyl acetate [19].

A more advanced method that is derived from LLE is high-speed counter-current chromatography (HSCCC), which is a high-performance liquid–liquid isolation method that lacks solid support resins and shows excellent sample recovery [20]. Therefore, HSCCC is widely used in natural product isolation and purification [21]. In some of our previous reports, this method was successfully applied to the preparative separation of a variety of marine natural products [22,23,24,25]. In this study, a new method was developed for efficient purification of baf A1 from *S. lohii* orf2&3 crude extract by applying a three-phase solvent system LLE followed by HSCCC with a two-phase solvent system (*n*-hexane–acetonitrile–water).

## 2. Results

### 2.1. Selection of a Three-Phase Solvent System for Extraction

**Figure 1:**
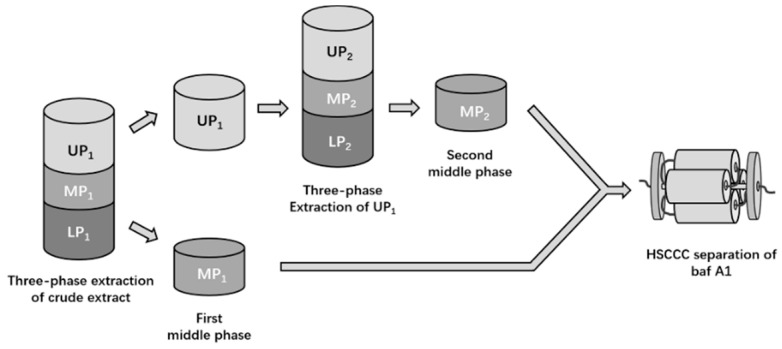
Flowchart of experimental design for baf A1 isolation and purification.

The overall experimental design for baf A1 isolation and purification is presented in Figure 1. Among several tested mixed solvent systems (Table 1), a few three-phase systems were observed in the mixtures with ratios ranging from 6:4:5:5 to 8:2:5:5, (*n*-hexane–ethyl acetate–acetonitrile–water, *v*/*v*/*v*/*v*). These three-phase mixtures were then tested to determine which could be most optimal to separate soybean oil, baf A1, and hydrophilic impurities by polarity. As shown in Table 1, the solvent system of 7:3:5:5 possessed higher baf A1 recovery in the MP (84.4%) than that of 8:2:5:5 (82.5%). Moreover, the solvent system ratio of 6:4:5:5 required much longer settling time, which would be inefficient or problematic in a large-scale process.

Next, we measured the distribution of soybean oil in different three-phase solvent systems in which only the solvent and soybean oil (without any crude extract) were mixed. As shown in Table 2, the concentration of oil in the MP of the solvent system of 7:3:5:5 or 8:2:5:5 was very low, demonstrating that over 96% of oil was removed from this partition. Although the solvent system of 8:2:5:5 showed a slightly better oil elimination effect than that of 7:3:5:5, the latter was selected for baf A1 enrichment due to its slightly higher recovery of baf A1 in the MP (Table 1). After a single extraction of the crude extract, the MP contained 84.4% of baf A1 and only 0.5% of the total soybean oil, as calculated from the data in Table 2. After the second repetition of three-phase liquid extraction, a total of 96.4% of baf A1 was recovered, and 99% of soybean oil and the majority of polar impurities were removed.

### 2.2. Determination of Partition Coefficient Values (K_D_) and HSCCC Separation

Determining partition coefficient values (K_D_) is a critical step in planning for HSCCC separation. According to the golden rules reported by Ito [20], an ideal two-phase solvent system should yield a suitable K_D_ value in the range from 0.5 to 2. In addition, short settling times (< 20 s) and high stationary-phase retention are required for good results in HSCCC.

**Figure 2:**
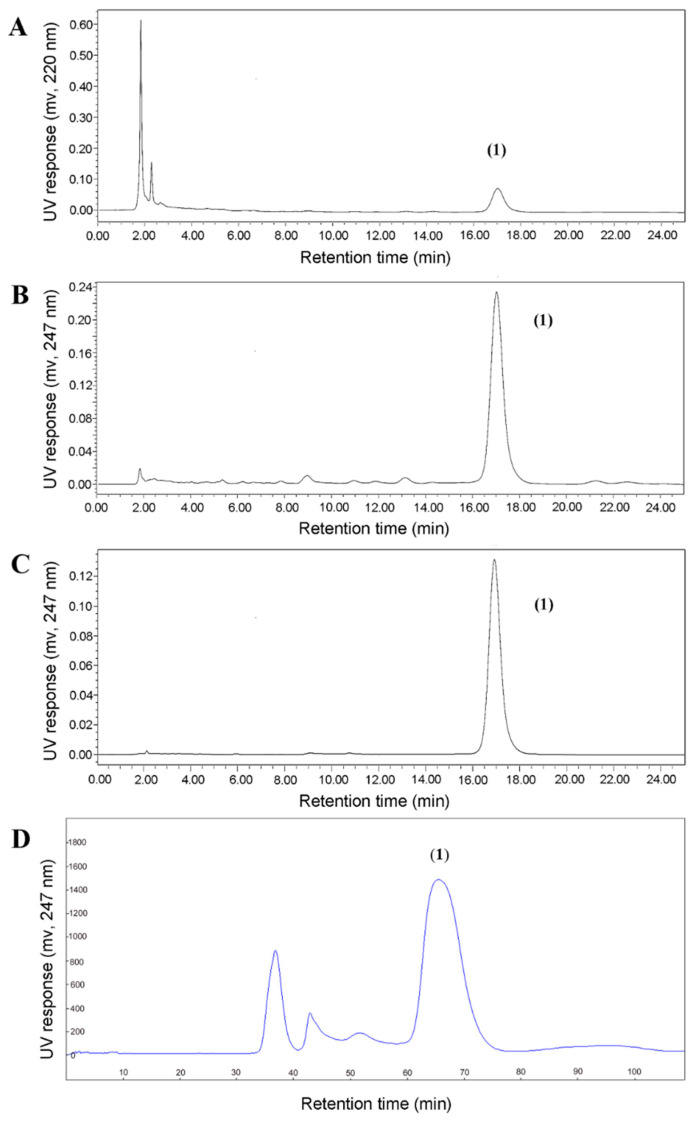
High-performance liquid chromatography (HPLC) chromatograms. (**A**) A crude extract sample from *S. lohii*; detection, 220 nm. (**B**) The same crude extract sample from *S. lohii*; detection, 247 nm. (**C**) The high-speed counter-current chromatography (HSCCC) fraction of baf A1; detection, 247 nm. Conditions: column, YMC-Pack C_18_ column (150 × 4.6 mm inner diameter (ID), 5 μm); column temperature, 25 °C; mobile phase, methanol and water in the isocratic elution mode (acetonitrile 80%, 25 min); flow rate, 0.8 mL/min; detection, 247 nm. (**D**) A representative HSCCC chromatogram of the enriched sample using *n*-hexane–acetonitrile–water (15:8:12, *v*/*v*/*v*). Conditions: stationary phase, lower phase; flow rate, 3.0 mL/min; revolution speed, 900 rpm; sample amount, 480.2 mg; separation temperature, 25 °C; detection wavelength, 247 nm; retention of the stationary phase: 62%.

Accordingly, a number of two-phase solvent systems composed of different ratios of *n*-hexane–acetonitrile–water were evaluated. As shown in Table 3, a smaller ratio of acetonitrile to water led to a larger K_D_ value of baf A1 in an acceptable range, and the solvent system of 15:8:12 was ultimately selected. Given the K_D_ value of 0.84, the HSCCC method was established with a flow rate of 3 mL/min, revolution speed of 900 rpm, and 62% retention rate of stationary phase. Under these conditions, 77.4 mg of purified baf A1 was obtained from a single HSCCC run in under 90 min using 480.2 mg of three-phase liquid partitioning enriched extract (Figure 2D).

### 2.3. Structural Identification of baf A1 after HSCCC

Based on HPLC analysis (Figure 2C), 77.4 mg of baf A1 was isolated with purity >95% from 480.2 mg of the enriched extract sample using HSCCC (Figure 2D). The overall recovery of baf A1 was 95.7%. The structural identification was carried out using electrospray ionization (ESI)-MS, as well as ^1^H- and ^13^C-NMR (Figures S1 and S2, Supplementary Materials). The results are summarized as follows, which matched literature values for baf A1 and our data obtained from a previous research project [26].

Compound (**1**) in Figure 2, white powder, ESI-MS (*m/z*): 645.26 [M + Na]^+^, ^1^H-NMR (500 MHz, CDCl_3_) *δ*: 6.68 (1H, s, H-3), 6.51 ((1H, dd, *J* = 15.01, 10.63 Hz, H-12), 5.81 (1H, d, *J* = 10.66 Hz, H-11), 5.77 (1H, d, *J* = 9.12 Hz, H-5), 5.54 (1H, d, *J* = 1.97 Hz, 19-OH), 5.16 (1H, dd, *J* = 15.07, 9.34 Hz, H-13), 4.95 (1H, dd, *J* = 8.68, 1.35 Hz, H-15), 4.65 (1H, d, *J* = 4.03 Hz, 17-OH), 4.13 (1H, ddd, *J* = 10.81, 4.21, 2.03 Hz, H-17), 3.88 (1H, t, *J* = 9.00 Hz, H-14), 3.69 (1H, m, H-21), 3.63 (3H, s, 2-OCH_3_), 3.49 (1H, dd, *J* = 10.24, 2.23 Hz, H-23), 3.29 (1H, d, *J* = 6.82 Hz, H-7), 3.24 (3H, s, 14-OCH_3_), 2.54 (1H, m, H-6), 2.30 (1H, dd, *J* = 11.90, 4.73 Hz, H-20), 2.15 (1H, m, H-16), 2.13 (1H, m, H-9), 1.99 (3H, d, *J* = 1.22 Hz, H-26), 1.95 (1H, m, H-9), 1.94 (3H, s, H-29), 1.90 (1H, m, H-8), 1.86 (1H, m, H-24), 1.77 (1H, m, H-18), 1.62 (1H, s, 7-OH), 1.32 (1H, m, H-22), 1.15 (1H, td, *J* = 11.57, 2.09 Hz, H-20), 1.07 (3H, d, *J* = 7.05, H-27), 1.04 (3H, d, *J* = 7.19 Hz, H-31), 0.94 (3H, d, *J* = 2.90 Hz, H-32), 0.93 (3H, d, *J* = 2.83 Hz, H-28), 0.90 (3H, d, *J* = 6.86 Hz, H-25), 0.83 (3H, d, *J* = 6.86 Hz, H-30), 0.77 (3H, d, *J* = 6.80 Hz, H-33); ^13^C-NMR (125 MHz, CDCl_3_) δ: 167.5 (C-1), 143.1 (C-10), 142.9 (C-5), 141.4 (C-2), 133.7 (C-3), 133.1 (C-12), 133.1 (C-4), 127.3 (C-13), 125.4 (C-11), 99.0 (C-19), 82.3 (C-14), 81.3 (C-7), 75.9 (C-23), 71.1 (C-21), 70.7 (C-17), 60.1 (2-OCH_3_), 55.6 (14-OCH_3_), 43.6 (C-20), 42.2 (C-18), 41.3 (C-9), 41.1 (C-22), 40.1 (C-8), 37.3 (C-37.3), 36.8 (C-36.8), 28.0 (C-24), 21.7 (C-21.7), 21.3 (C-25), 20.3 (C-29), 17.4 (C-27), 14.4 (C-33), 14.1 (C-26), 12.2 (C-32), 9.9 (C-30), 7.2 (C-31).

### 2.4. Comparison of HSCCC and Semi-Preparative HPLC for the Preparative Separation of baf A1

The efficiency of HSCCC and HPLC for preparative separation of baf A1 from previous enrichment was assessed. The result presented in Table 4 shows that HSCCC could achieve higher productivity of baf A1 with lower organic solvent consumption compared with semi-preparative HPLC. Furthermore, HSCCC is an affordably scalable method with industrial applications that are much more affordable than preparative HPLC due to associated solid support and instrumental costs.

## 3. Discussion

Oil is often added to the fermentation broths of actinomycetes to enhance the production of polyketide natural products. However, the subsequent extraction of the broth by organic solvent typically results in a crude extract with a high concentration of oil, which is undesirable and introduces significant difficulties in downstream processing and chromatographic steps for the purification of target compounds. This study describes a novel and efficient method for the rapid enrichment and separation of baf A1 from oils and crude extract of marine *S. lohii* fermentation broth. Compared with classical liquid two-phase extractions and column chromatography, this method is based on a three-phase solvent system capable of very efficiently removing residual soybean oil and most other impurities in the crude extract. Furthermore, rapid separation of baf A1 with high yield and purity was achieved from the resulting enriched partition fraction using HSCCC. The results of the present study indicate that liquid–liquid extraction with a three-phase solvent system significantly simplifies the enrichment process of natural products from a complex matrix. It was also demonstrated that HSCCC is a powerful technique for the rapid preparative isolation and purification of natural products, which is in line with many other reports about this technique. The established strategy for enrichment and separation may provide a useful approach to fill the gap between large-scale natural product fermentation and purification for producing the sample supply required for in-depth pharmacological investigations and drug development, which can be applied to baf A1, as well as other target molecules.

## 4. Materials and Methods

### 4.1. Reagents and Materials

All organic solvents used for three-phase LLE and two-phase HSCCC were of analytical grade and purchased from Huadong Chemicals (Hangzhou, China). Chromatographic-grade solvents used for HPLC analyses were purchased from Anpel Laboratory Technologies (Shanghai, China).

### 4.2. Crude Extract

A single colony of *S. lohii* orf2&3 was inoculated into 30 mL of 2× YT medium at a 1:10 ratio and cultured on shakers at 220 rpm, 28 °C for 2 d. Then, 3 mL of seed culture was transferred into each 30 mL of fermentation medium (20 g of glucose, 20 g of soybean flour, 60 g of soybean oil, 2 g of NZ-amine, 1.5 g of corn syrup, 1 g of yeast extract, 8 g of NaNO_3_, 8 g of CaCO_3_, 6 g of (NH_4_)_2_SO_4_, 5 g of NaCl, and 0.3 g of K_2_HPO_4_ per liter) at 28 °C, 250 rpm for another 7 d. Finally, a total of 10 L of fermentation culture was collected and extracted with EtOAc, and a crude extract (4.9 g) was recovered after concentration under vacuum.

### 4.3. Apparatus

HSCCC separation was conducted using a TBE-300C high-speed counter-current chromatograph (Tauto Biotech Co. Ltd., Shanghai, China) equipped with three serially connected polytetrafluoroethylene (PTFE) multi-layer coils (inner diameter (ID) 2.6 mm, total volume 300 mL) and a 20-mL sample loop. The separation temperature was adjusted using an HX 1050 constant-temperature circulating implement (Beijing Boyikang Lab Instrument, Beijing, China). An S-1007 constant flow pump (Shenyitong Tech & Exploitation, Beijing, China) was applied to pump both the stationary and the mobile phases. The outlet effluent was continuously monitored using a C-635 ultraviolet (UV) photometer (Buchi, Flawil, Switzerland). The HSCCC separation data collection and analysis was carried out using N2000 chromatography workstation (Zhejiang University, Hangzhou, China). HPLC analysis was conducted on a Waters Alliance 2695 with a Waters model 2996 diode array detector and was controlled by a Waters Empower System (Waters Co., Milford, MA, USA). A Waters 600 system (Waters, MA, USA) equipped with a 5-mL injection system and a Waters 2996 photodiode array detector were used for semi-preparative isolation.

Compound characterization was performed using a Thermo TSQ Quantum Access spectrometer with an ESI interface (Thermo Fisher Scientific, San Jose, CA, USA) and a Bruker AVANCE-500 NMR spectrometer using standard pulse programs (Bruker, Fällanden, Switzerland).

### 4.4. Selection of Three-Phase Solvent System for Extraction

Small volumes of *n*-hexane, ethyl acetate, acetonitrile, and water were mixed in different ratios to form a 20-mL mixture in a serum bottle, and the volume of each phase was recorded. The crude extract of *S. lohii* orf2&3 fermentation culture (100 mg) was dissolved completely in each three-phase solvent system at room temperature. The three phases were separated using a glass funnel; then, the MP was concentrated at 40 °C under reduced pressure to afford the first MP extract (MP_1_). The UP extract (UP_1_) was re-dissolved in a newly prepared three-phase solvent system to produce the second MP extract (MP_2_), which improved recovery of the target compound, baf A1. The two MPs were combined and dried to afford a baf A1-enriched fraction (Figure 1). The concentrations of baf A1 in different phases were analyzed by HPLC.

To analyze the distribution of soybean oil in the three-phase extraction system, 5 mL of soybean oil was mixed vigorously with a total of 40 mL of the three-phase solvent system composed of *n*-hexane–ethyl acetate–acetonitrile–water in a separatory funnel at room temperature. Each phase was separated and, after settlement, concentrated under vacuum. The volume and weight of each phase was measured before and after solvent evaporation. As a control, 5 mL of soybean oil was treated in the same way for partial evaporation and weight measurement. Each test was performed in triplicate.

### 4.5. Preparation of Enriched Example

Firstly, 4.9 g of crude extract of *S. lohii* orf2&3 fermentation was completely dissolved in the selected three-phase solvent system. The first UP was re-extracted with the identical solvent system. The first and second MPs were combined and concentrated, yielding 480.2 mg of a baf A1-enriched fraction.

### 4.6. Selection of Partition Coefficient Values (K_D_)

Solvent systems composed of *n*-hexane–acetonitrile–water were used for the testing of partition coefficient values (K_D_). The method used to measure this was as follows: firstly, a small amount (10 mg) of the enriched baf A1 fraction was added into a 2-mL Eppendorf tube containing pre-equilibrated two-phase solvents. The equilibration of target compound between two phases was conducted by shaking thoroughly for 3 min. Each settled phase was then passed through a 0.3-μm nylon membrane filter and analyzed by HPLC. The K_D_ values were calculated using the equation K_D_ = A_1_/A_2_, wherein A_1_ and A_2_ represent the peak areas of target compound in upper and lower phase, respectively.

### 4.7. Preparation of the Two-Phase Solvent System and Sample Solution

In the present study, a two-phase solvent system consisting of *n*-hexane–acetonitrile–water (15:8:12, *v*/*v*/*v*) was used for the HSCCC isolation. The mixture of this solvent system (2 L in total) was added to a separation funnel and vigorously shaken and equilibrated at room temperature. The two phases were separated and degassed by sonication for 15 min shortly before use. For HSCCC isolation, the sample solution was prepared by dissolving 480.2 mg of enriched baf A1 in 15 mL of the pre-equilibrated two-phase solvent system.

### 4.8. HSCCC Separation

In this study, the organic UP of the two-phase solvent system was selected to be the stationary phase and the aqueous LP was selected as the mobile phase. The HSCCC procedure was as follows: the multilayer coiled column was completely filled with the organic UP. Then, the aqueous LP was pumped into the column from head to tail at a flow rate of 3.0 mL/min, while the column was smoothly rotated at 900 rpm. After the hydrodynamic equilibrium of system was established, as indicated by the mobile phase emerging from the column outlet, the prepared sample solution (15 mL) was manually loaded through the injection valve. The effluent from the column was continuously monitored using a UV detector at the wavelength of 247 nm, and this was collected automatically in 5 min fractions of 15 mL in individual test tubes. During the HSCCC isolation process, the column temperature was 25 °C. According to the elution UV chromatogram, collected peak fractions were analyzed by HPLC for combination of multiple test tubes or fraction pooling. After separation, the solvent in the column was replaced with nitrogen for protected long-term storage.

### 4.9. HPLC Analysis, Semi-Preparative HPLC Purification of baf A1 in Fractions from HSCCC Separation, and Compound Identification by MS and NMR

HPLC analyses of crude sample and HSCCC peak fractions were performed using a Waters Alliance 2695 liquid chromatography system, equipped with a reverse-phase YMC-Pack C_18_ column (150 × 4.6 mm ID, 5 μm, YMC Co. Ltd., Tokyo, Japan) at 25 °C. The purity of baf A1 was determined by ^1^H-NMR spectroscopy and an HPLC external standard method. HPLC-purified baf A1 was selected as external standard. For all HPLC analyses, the mobile phase was acetonitrile and water with a flow rate of 0.8 mL/min in isocratic elution mode (acetonitrile 80%, 25 min), and the eluent was evaluated by UV detection at 247 nm. A Waters Alliance 2695 liquid chromatography system equipped with a larger YMC-Pack C_18_ column (250 × 10 mm ID, 5 μm, YMC Co. Ltd., Tokyo, Japan) was used for semi-preparative purification of baf A1 from the previous HSCCC enriched fractions. The flow rate was 2 mL/min, and acetonitrile and water (acetonitrile 80%, 30 min) was used as the mobile phase in isocratic elution mode. The wavelength of UV detection and the column temperature were identical to those of the analytical HPLC protocol. The compound identification of baf A1 in HSCCC peak fractions and semi-preparative HPLC fractions was conducted by ESI-MS, as well as ^1^H-NMR and ^13^C-NMR analyses.

## Figures and Tables

**Table 1:** Phase partitioning of baf A1 in different three-phase solvent systems (*n*-hexane–ethyl acetate–acetonitrile–water). UP—upper phase; MP—middle phase; LP—lower phase.

Volume Ratio	Phase Formation	Phase Ratio (%)	Baf A1 Distribution (UP/MP/LP %)	System Settling Time (Second)
3:7:5:5	2	68.6/31.4	/	/
5:5:5:5	2	67.6/32.4	/	/
6:4:5:5	3	31.4/34.3/34.3	15.1/84.9/0	>180
7:3:5:5	3	38.9/25/36.1	15.6/84.4/0	50
8:2:5:5	3	45.7/17.1/37.2	15.9/82.5/1.56	<20
9:1:5:5	2	47.2/52.8	/	/

**Table 2:** Distribution of soybean oil in different solvent systems upon a single extraction of the crude extract.

Solvent Systems	UP (g)	MP (g)	Control (g) ^1^	Total Soybean Oil Elimination (%)
6:4:5:5	4.41	0.11	4.67	94.43%
7:3:5:5	3.94	0.02	4.10	96.09%
8:2:5:5	4.42	0.02	4.52	97.78%

^1^ 5 mL of soybean oil dried individually.

**Table 3:** Partition coefficient values (K_D_) of baf A1 in different solvent systems (*n*-hexane–acetonitrile water).

Volume Ratio	K_D_ (MP Extract)	Volume Ratio (UP/LP)
15:6:6	0.06	1.2/1
15:8:8	0.08	1/1
15:10:10	0.12	1/1.3
15:9:11	0.28	1.3/1
15:8:12	0.84	1.1/1
15:7:13	1.74	1/1.3
15:6:14	2.45	1/1.4

**Table 4:** Comparison of HSCCC and semi-preparative HPLC with regard to baf A1 separation.

	HSCCC	HPLC
Stationary phase	Upper phase *n*-hexane–acetonitrile–water (15:8:12, *v*/*v*/*v*)	YMC-Pack C_18_ column (250 × 10 mm ID, 5 μm)
Mobile phase	Lower phase	Acetonitrile–water (80:20, *v*/*v*)
Sample capacity per run (mg)	480	5
Run time (min)	90	30
Productivity (mg/min)	0.81	0.03
Purity of isolated compound	>95%	98%
Organic solvent consumption (L/g)	2.29	68.57

## Supplementary Materials

The following are available online at https://www.mdpi.com/1660-3397/18/6/332/s1: Figure S1. ^1^H-NMR (500 MHz, CDCl_3_) spectrum of bafilomycin A1; Figure S2. ^13^C-NMR (125 MHz, CDCl_3_) spectrum of bafilomycin A1.
