# Supplementary material for: Efficient Preparation of Bafilomycin A1 from Marine Streptomyces lohii Fermentation Using Three-Phase Extraction and High-Speed Counter-Current Chromatography
Source: Mar Drugs. 2020 Jun 25;18(6):332. doi: 10.3390/md18060332 (PMC7345786; doi:10.3390/md18060332)
Supplement: Supplementary file 1 [file marinedrugs-18-00332-s001.pdf]

Supplementary Materials

Efficient Preparation of Bafilomycin A1 from Marine *Streptomyces lohii* Fermentation Using Three-Phase Extraction and High-Speed Counter-Current Chromatography

Ye Yuan [1,2], Xiaoping He [1], Tingting Wang [1], Xingwang Zhang [3], Zhong Li [4,5], Xiaoqing Xu [6], Weiyan Zhang [1], Xiaojun Yan [2,*], Shengying Li [3,*], Shan He [1,*]

[1] Li Dak Sum Yip Yio Chin Kenneth Li Marine Biopharmaceutical Research Center, College of Food and Pharmaceutical Sciences, Ningbo University, Ningbo, Zhejiang 315800, China; 23yuanye@163.com (Y.Y.); 18892628896@163.com (X.H.); 18251965896@163.com (T.W.); zhangweiyan13@126.com (W.Z.)

[2] School of Marine Science, Ningbo University, Ningbo, Zhejiang 315211, China

[3] State Key Laboratory of Microbial Technology, Shandong University, Qingdao, Shandong 266237, China; zhangxingwang@sdu.edu.cn (X.Z.)

[4] Shandong Provincial Key Laboratory of Synthetic Biology, and CAS Key Laboratory of Biofuels at Qingdao Institute of Bioenergy and Bioprocess Technology, Chinese Academy of Sciences, Qingdao, Shandong 266101, China; lizhong@qibebt.ac.cn (Z.L.)

[5] University of Chinese Academy of Sciences, Beijing 100049, China

[6] Rushan Hanwei Biological Science and Technology Co., Ltd., Rushan, Shandong 264502, China; xiaoqingxu@163.com (X.X.)

* Correspondence: yanxiaojun@nbu.edu.cn (X.Y.); lishengying@sdu.edu.cn (S.L.); heshan@nbu.edu.cn (S.H.); Fax: +86-574-87600458 (X.Y.); +86-574-87604388 (S.H.).

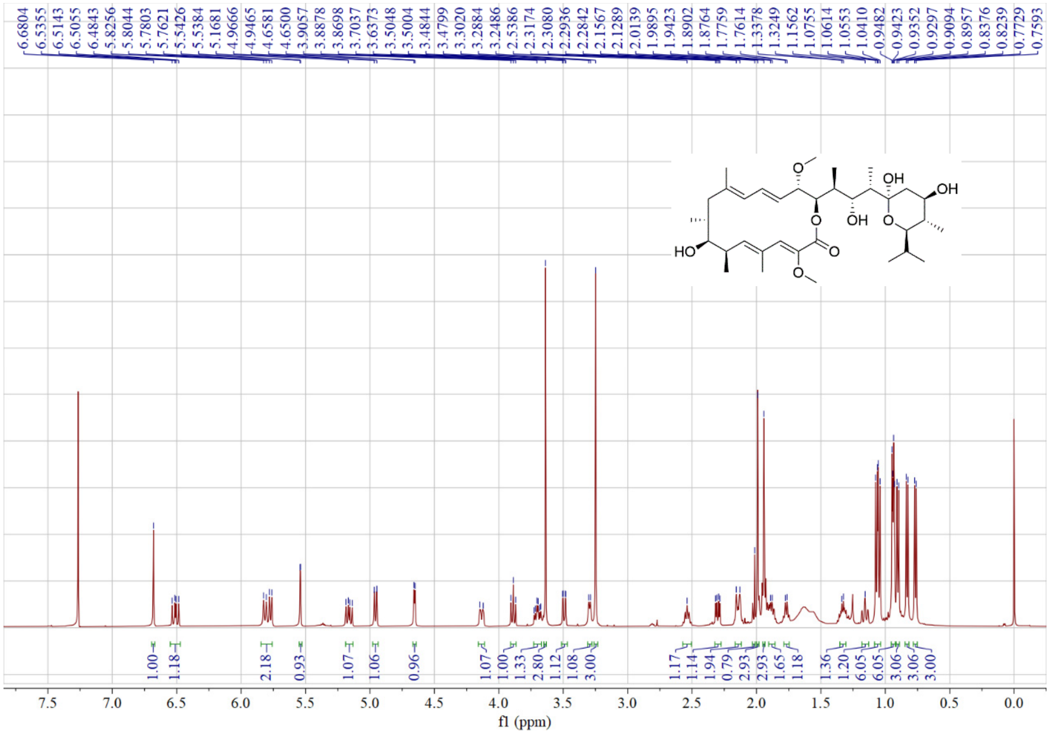

Figure S1. ^{1}H NMR (500 MHz, $CDCl_3$) spectrum of bafilomycin A1

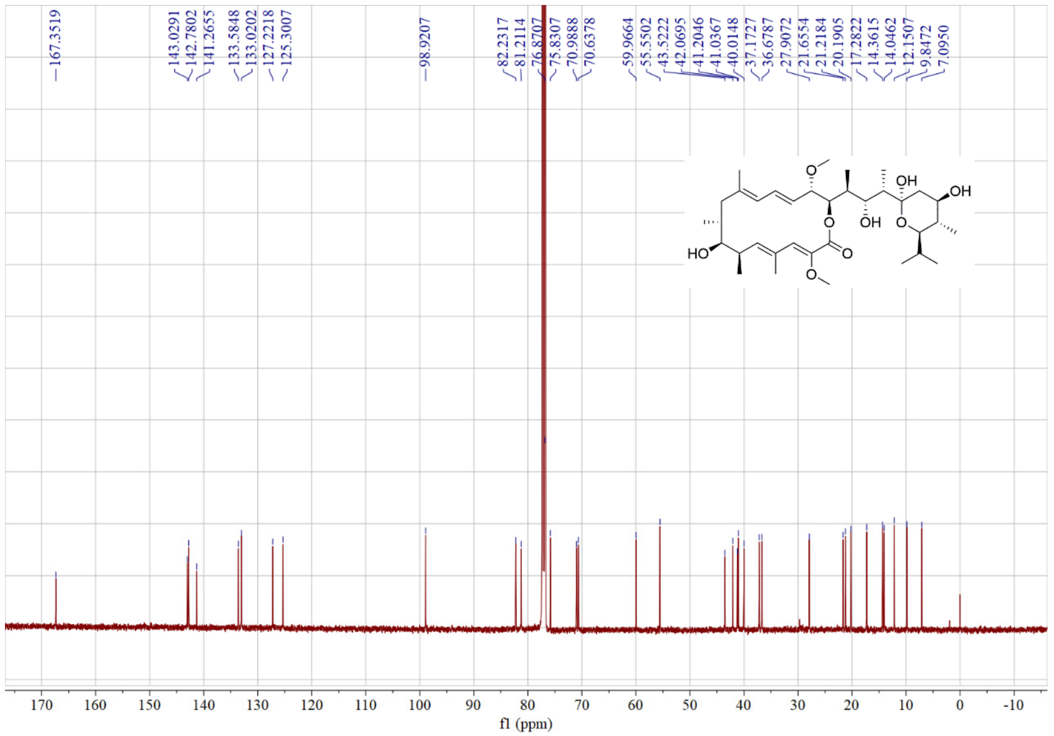

Figure S2. ^{13}C NMR (125 MHz, $CDCl_3$) spectrum of bafilomycin A1
